# Mantle cell lymphoma and its management: where are we now?

**DOI:** 10.1186/s40164-019-0126-0

**Published:** 2019-01-30

**Authors:** Abdullah Ladha, Jianzhi Zhao, Elliot M. Epner, Jeffrey J. Pu

**Affiliations:** 1 Upstate Cancer Center, State University Of New York Upstate Medical University, SUNY Upstate Cancer Center, Suite 331, CWB, 750 E. Adams Street, Syracuse, NY 13210 USA; 2 Penn State Hershey Cancer Institute, Pennsylvania State University College of Medicine, Hershey, PA 17033 USA; 3 Syracuse VA Hospital, State University of New York Upstate Medical University, Syracuse, NY 13210 USA

**Keywords:** Mantle cell lymphoma, Pathophysiology, Novel agents, Combination therapy, Clinical trial

## Abstract

Mantle cell lymphoma is a relatively new recognized hematological malignant disease, comprising of 2.5–6% non-Hodgkin’s lymphomas. The complexity of its clinical presentations (nodular pattern, diffuse pattern, and blastoid variant), variety in disease progression, and treatment response, make this disease a research focus to both experimental oncology and clinical oncology. Overexpression of cyclin D1 and chromosome t(11,14) translocation are the known molecular biomarkers of this disease. Mantle cell international prognostic index (MIPI), ki-67 proliferation index, and *TP53* mutation are emerging as the prognostic biomarkers. Epigenetic profile variance and *SOX11* gene expression profile correlate with treatment response. Over the years, the treatment strategy has been gradually evolving from combination chemotherapy to combination of targeted therapy, epigenetic modulation therapy, and immunotherapy. In a surprisingly short period of time, FDA specifically approved 4 drugs for treating mantle cell lymphoma: lenalidomide, an immunomodulatory agent; Bortezomib, a proteasome inhibitor; and Ibrutinib and acalabrutinib, both Bruton kinase inhibitors. Epigenetic agents (e.g. Cladribine and Vorinostat) and mTOR inhibitors (e.g. Temsirolimus and Everolimus) have been showing promising results in several clinical trials. However, treating aggressive variants of this disease that appear to be refractory/relapse to multiple lines of treatment, even after allogeneic stem cell transplant, is still a serious challenge. Developing a personalized, precise therapeutic strategy combining targeted therapy, immunotherapy, epigenetic modulating therapy, and cellular therapy is the direction of finding a curative therapy for this subgroup of patients.

## Introduction

In 1970s, investigators observed a histologically distinctive subtype of non-hodgkin lymphoma (NHL), which appeared intermediate between well-differentiated (small lymphocytic lymphoma) and poorly differentiated (small cleaved cell lymphoma) and resembled centrocytes of reactive germinal center. This new subtype of NHL was called intermediate lymphocytic lymphoma [1–3]. Later investigators identified that this subtype lymphoma originated from mantle zones of secondary follicles and express B cell markers but different from follicular lymphoma [4–6]. It was called mantle cell lymphoma and was further stratified into nodular, diffuse and mantle zone subtypes. Mitotic activity, blastic morphology and peripheral blood involvement at diagnosis were recognized as poor prognostic indicators [7, 8].

MCL comprises of 2.5–6% of NHLs [9, 10]. MCL usually have either nodular or diffuse pattern of growth. Approximately 20% MCL cases show blastoid morphology. The MCL cells express surface immunoglobulins, including Ig M and Ig D, CD5, CD19, and CD22; but not CD3, CD23, CD10 and CD11c. Fluorescent in situ hybridization (FISH) reveals translocation t(11; 14) in almost all MCL cells [11]. Mantle cell lymphoma international prognostic index (MIPI) score can divide patients into low, intermediate and high risk groups. Low risk group shows a 5 year overall survival (OS) rate of 60%, intermediate risk group has median OS of 51 months and high-risk group has median OS of 29 months [12]. Ki-67 proliferation index is a prognostic biomarker independent of MIPI score and predicts survival in patients receiving high dose chemotherapy and autologous stem cell transplant (ASCT) [13]. Molecular marker like SOX11 is associated with aggressive phenotype [14].

The way to treat MCL has evolved over time, although indolent form of disease may be observed, patients with aggressive variant have different treatment options depending on age, performance status and possibility of bone marrow transplant. New advances including novel targeted therapy and immunotherapy have changed the landscape of treatment (Table 1).

**Table 1:**
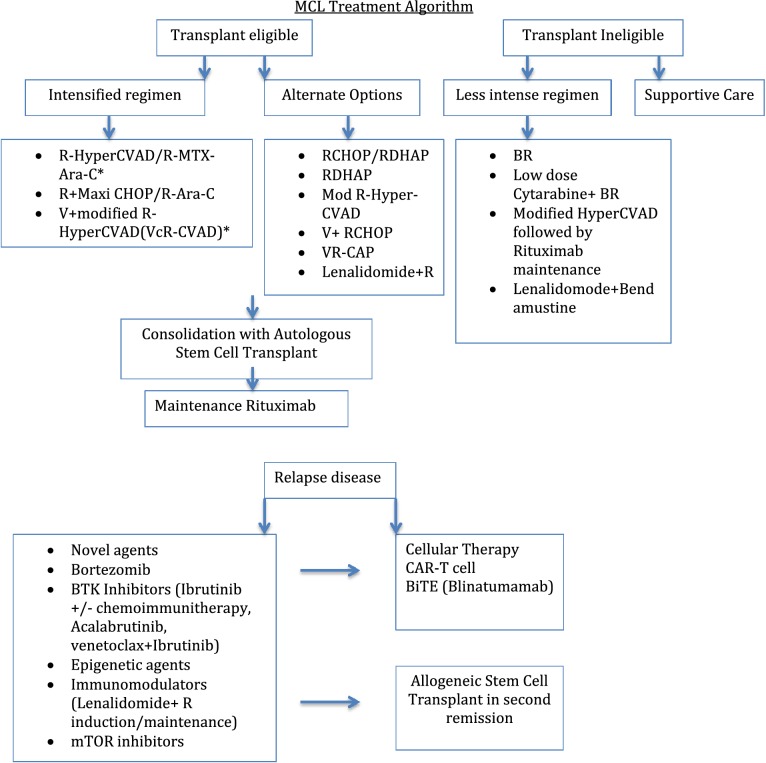
MCL treatment algorithm

R-HyperCVAD (Rituximab-Hyperfractionated cyclophosphamide, vincristine, Adriamycin and dexamethasone); Mod: modified; VcR-CVAD (bortezomib with modified R-HyperCVAD); CHOP (Cyclophosphamide, vincristine, doxorubicin and prednisone); RDHAP (Rituximab, dexamethasone, cytarabine and platinum); V (Bortezomib); VR-CAP (bortezomib, rituximab, cyclophosphamide, doxorubicin and prednisone); BTK: Bruton tyrosine kinase; mTOR: mechanistic target of rapamycin inhibitors; BiTE (Bi-specific T-cells Engager); CAR-T: chimeric antigen receptor-engineered T-cells

* Some trials did not include consolidation with ASCT

## Problem in MCL treatment

In late 1980s, Meusers et al. investigated if centrocytic lymphoma/mantle cell lymphoma was a curable entity. Patients were randomized to COP or anthracycline containing CHOP (cyclophosphamide, doxorubicin, vincristine and prednisone). There were no significant differences with respect to rates of remission induction, probability of progression free survival (PFS) and overall survival (OS). The median survival times for those patients were 33 months. This study suggested that conventional treatment options were not able to improve prognosis at that time [15].

Compared to other NHLs, MCL exhibits shorter durations of response, PFS and OS. In 1995, Teodorovic et al. analyzed two EORTC Lymphoma cooperative group trials and showed that patients with MCL in all grades had shorter duration of response and PFS compared to patients with other lymphomas, though the initial response rates were very similar [16]. Interestingly, adding rituximab to CHOP therapy improved the objective response rate (ORR) to 94–99% [17]. However, this high ORR did not translate into a PFS benefit. Furthermore, Lenz et al. in a GLSG (German Low Grade Lymphoma Study Group) study, showed that addition of rituximab in induction therapy didn’t improve PFS among ASCT patients either [18].

## Elderly and transplant ineligible patients

As patients with MCL tend to be elderly with comorbidities, most of the patients are not eligible for intensive induction therapies or ASCT. Bendamustine plus rituximab (BR) emerged as the combination therapy for this group of patients. STiL trial and BRIGHT study demonstrated better CR and improved PFS in comparison to RCHOP/RCVP therapy in elderly patients [19, 20].

Cytarabine has been proved an effective regimen in MCL intensive induction therapy. It was shown that adding low dose cytarabine (500 mg/m^2^) to BR in treating MCL patients, especially for elderly patients who were ineligible for ASCT, resulted in PET negative CR rate of 91%, PFS rate of 76% at median follow up of 35 months but at the cost of increased myelosuppression, especially thrombocytopenia [21].

Another less intense chemotherapy option for elderly patients was modified Hyper-CVAD (without methotrexate or cytarabine). In 2006, Kahl et al. reported the results of a phase II trial, treating patients with 4 to 6 cycles of modified Hyper-CVAD, followed by 2 years of rituximab maintenance [22]. This study showed overall response rate of 77%, CR rate of 64%, median PFS of 37 months and OS not reached. Two years of rituximab maintenance prolonged PFS rate.

## Intensive therapies

In 1998, Khouri et al. demonstrated that 4 cycles of Hyper CVAD/MTX-Ara-C regimen followed by stem cell transplant was superior to standard CHOP-like therapy in previously untreated patients (OS of 92% vs 56% and PFS of 72% vs 28%) [23]. In 2005, Romaguera et al. extended this inductive regimen to 6 to 8 cycles and added rituximab in a phase II trial to young and newly diagnosed aggressive MCL patients, and showed an improved response rate, PFS, and OS [24]. These studies suggested the promising role of intensified induction chemo-immunotherapy followed by transplantation in treating young patients with newly diagnosed aggressive MCL. In 2008, Geiseler et al. demonstrated that combination of intensified chemotherapy with immunotherapy in younger patients would not only yield better response rate but also resulted in an improved long term outcome in a Nordic lymphoma group (NLG) protocol (MCL-2) [25]. Wisconsin Oncology Network trial combining bortezomib with Hyper-CVAD induction regimen without transplant and demonstrated CR of 95% [26, 27]. However, intensified chemotherapy regimen accompanied a significant treatment related toxicity profile including grade 4 neutropenia, thrombocytopenia, severe mucositis, and serious infections.

Efficacy of autologous transplantation was shown to improve with Ara-C containing myeloablative therapy and rituximab maintenance therapy. MCL Younger trial of European mantle cell lymphoma network (MCL net) compared 6 courses of R-CHOP followed by myeloablative radiochemotherapy and ASCT to alternating course of three cycles of CHOP and three cycles of DHAP (dexamethasone, cytarabine and cisplatin or platinum) plus rituximab followed by high dose Ara-C containing myeloablative regimen and ASCT. CR rate and time to treatment failure was significantly higher in arm containing high dose Ara-C followed by ASCT, 36% vs. 25% and 88 months vs. 46 months respectively [28]. In a 2012 update, MCL net showed improved survival with longer median follow up of 27 months in treatment arm that included Ara-C followed by ASCT (not reached vs. 82 months) [29].

Efficacy of rituximab as maintenance therapy after ASCT was investigated by Le Gouill et al. (LyMa trial) [30]. In this trial newly diagnosed patients received R-DHAP based induction, followed by conditioning regimen with BEAM (Carmustine, etoposide, cytarabine and melphalan) and ASCT. Patients who responded went on to receive 3 years of maintenance rituximab. In this trial, patients in rituximab maintenance had 60% reduction of the progression risk and 50% reduction in the death risk. This was the first trial that showed maintenance rituximab after ASCT prolongs EFS (event free survival), PFS and OS.

The above-mentioned trials have established the role of autologous stem cell transplant in patients who respond to first line induction chemo-immunotherapy. Role of allogeneic stem cell transplant in relapsed patients who fail autologous stem cell transplant as been studied as well. Using non-myeloablative conditioning for relapsed MCL patients, investigators at MD Anderson showed a 5 year overall survival and progression free survival of 49% and 37%, respectively [31]. Another retrospective study, using reduced intensity conditioning for relapsed MCL patients, showed median overall survival of 62 months with 32% treatment related mortality in 3 years [32].

## Novel agents (Table 2)

### Bortezomib

Bortezomib (Valcade), a proteasome inhibitor, has shown efficacy as monotherapy, in relapsed MCL patients with response rate and CR rate reported as 33% and 8% respectively [33]. When combined with R-CHOP in frontline setting, bortezomib has shown ORR of 81% to 91%, with CR of 64% and median PFS of 23 months [34]. Also in first line setting, combination of Bortezomib with rituximab, cyclophosphamide, adriamycin and prednisone (VR-CAP) had resulted in better median PFS in comparing with RCHOP, 24.7 months vs. 14.4 months [35]. Bortezomib maintenance therapy after Bortezomib-RCHOP induction showed that it not only was well tolerated but also improved CR rate to 83% and median PFS to 29.5 months [36].

**Table 2:** Clinical trials have been conducted targeting various parts of MCL pathophysiology

Target	Study	Setting	Phase	Regimen	ORR	CR	PFS	OS
1	*Proteasome inhibitor*							
	Fisher et al. [33]	Relapsed	I/II	V	33%	8%	NA	NA
	Ruan et al. [34]	Front line	I/II	V + RCHOP	91%	64%	23 months	NR
	Till BG et al. [36]	Front line	II	V + RCHOP	83%	57%	29.5 months	NR
	Chang et al. [37]	Front line	II	V + Modified R-HyperCVAD (VcR-CVAD) and MR for 5 years	90%	77%	8.14 years	NR
2	*Bruton’s tyrosine kinase inhibitor*							
	Rule et al. [40]	Relapsed/refractory	PA	Ibrutinib monotherapy	69.7%	26.5%	13 months	26.7 months
	Wang et al. [41]	Relapsed/refractory	II	Ibrutinib + R	88%	44%	75% (12 months)	85.5% (12 months)
	Maddocks et al. [42]	Relapsed or new MCL	I/Ib	Ibrutinib + BR	94%	76%	NA	NA
	Wang et al. [44]	Relapsed	II	Acalabrutinib	81%	40%	67% (12 months)	87% (12 months)
	Walter et al. [45]	Relapsed	I	ONO/GS-4059	92%	46%	NA	NA
	Tam et al. [46]	Relapsed	II	Ibrutinib+venetoclax	71%	62%	NA	NA
3	*Epigenetic agents*							
	Pu et al. [48]	New or relapsed/refractory MCL	I/II	Cladribine + R + V	85%	77%	NR	NR
	Spurgeon et al. [47]	New or relapsed MCL, CLL, NHL	II	Vorinostat + Cladribine + R	97% (untreated MCL)	80% (untreated MCL)	70.7% (24 months)	86% (24 months)
	Puvvada et al. [49]	New or relapsed MCL and indolent NHL	II	Cladribine + R + V	100%	50%	82% (24 months)	91% (24 months)
4	*Immunomodulatory agent*							
	Goy et al. [50]	Relapsed	II	Len monotherapy	28%	7.5%	4 mon	19 mon
	Habermann et al. [51]	Relapsed	II	Len monotherapy	53%	20%	5.6 mon	NA
	Witzig et al. [52]	Relapsed	II	Len monotherapy	42%	21%	5.7 mon	NA
	Wang et al. [54]	Relapsed/refractory	I/II	Len + R	57%	36%	11.1 mon	24.3 months
	Ruan et al. [55, 56]	First line	II	Len + R	92%	64%	Not reached	97% (2 years OS)
	Morrison et al. [53]	Relapsed/refractory	II	Len + V	39.6%	15.1%	7 months	26 months
	Albertsson-Lindblad et al. [57]	First line	I/II	Len + BR	91%	78%	42 months	53 months
5	*mTOR kinase inhibitors*							
	Hess et al. [58]	Relapsed/refractory	III	Temsirolimus vs. investigator’s choice	6–22%	0–2%	4.8–3.4 months	12.8–10 months
	Witzig et al. [59]	Relapsed	II	Temsirolimus monotherapy	30%	3%	6.5 months	12 months
	Ansell et al. [60]	Relapsed	II	Temsirolimus monotherapy	41%	3.7%	6 months	NA
	Wang et al. [62]	Refractory to V	II	Everolimus monotherapy	8.6%	0%	4.4 months	16.9 months
	Hess et al. [61]	Relapsed	I	Temsirolimus + BR	92%	45%	NA	NA
6	*CART and BiTE*							
	Abramson et al. [66]	Relapse/refractory	Pivotal trial	Lisocabtagene maraleucel (JCAR017)	72% (NHL)	52% (NHL)	NA	NA
	ZUMA-2 [68]	Relapsed/refractory	II	Axicabtagene ciloleucel	NA	NA	NA	NA
	Goebeler et al. [69]	Relapsed/refractory	I	Blinatumomab	71%	NA	NA	NA
	Dufner et al. [70]	Relapsed/refractory	I	Blinatumomab	NA	NA	204 days	1560 days
	Budde et al. [71]	Relapsed/refractory	I	Mosunetuzumab	41%	NA	NA	NA

*V* Bortezomib; *R* rituximab; *RCHOP* rituximab, cyclophosphamide, doxorubicin, vincristine and prednisone; *R-HyperCVAD* rituximab, hyperfractionated cytarabine, vincristine, doxorubicin and dexamethasone; *MR* maintenance rituximab; *BR* Bendamustine and rituximab; *Len* Lenalidomide; *CART* Chimeric antigen receptor-engineered T-cells; *BiTE* Bi-specific T-cells Engager; *NA* not available; *NR* not reached; *PA* pooled analysis

Combination of bortezomib with intensive therapy has been shown to be safe [37]. Addition of bortezomib to modified R-HyperCVAD or VcR-CVAD (no vincristine on day 11 and no alternating doses of methotrexate/cytarabine) made long-term remission possible. Combined maintenance therapy with rituximab and bortezomib in a post-transplant setting was also shown to result in 2 years DFS and OS of 93.8% and 92.3% respectively [38].

### Bruton’s tyrosine kinase (BTK) inhibitors

Early studies in relapsed setting showed that Ibrutinib, a Bruton’s tyrosine kinase inhibitor resulted in response rate and CR of 77% and 33% respectively [39]. In a pooled analysis of Ibrutinib treatment in relapsed and refractory MCL, CR was achieved in 26.5% patients, median PFS was 13 months, PFS with one prior line of chemotherapy was 33.6 months and median OS was 26.7 months [40]. It has been combined with rituximab, bendamustine and RCHOP in treating naïve and refractory cases [41–43]. These combinations have resulted in higher responses. When combined with rituximab in relapsed setting, it showed objective response rate and CR of 88% and 44% respectively. Important adverse events noted were fatigue, myalgia, grade 3 nasal bleeding, 12% of patients had grade 3 atrial fibrillation and one patient had grade 3 leukocytosis. In combination with bendamustine and rituximab in phase I/Ib study, 94% patients showed objective response and 76% showed CR. Main adverse events were due to cytopenias and rashes (25%). Early phase study of Ibrutinib in combination with R-CHOP, in treatment naïve setting, showed overall response rate of 94% with grade 4 toxicity of neutropenia.

The emergence of resistance to Ibrutinib has led to development of more specific second generation BTK inhibitors including acalabrutinib (ACP-196) and ONO/GS-4059. A recently published phase II study of acalabrutinib in relapsed/refractory showed 81% overall response rate and 40% CR rate. This new BTK inhibitor is less toxic in phase I trial and better tolerated, it does not cause increased atrial fibrillation and bleeding events were noted in Ibrutinib trials [44, 45].

Recently, combination of Ibrutinib and venetoclax (direct inhibitor of BCL2) in patients with refractory disease showed overall response rate of 71% at 16 weeks as assessed by PET scan. Absence of minimal residual disease was documented in 67% patients according to flow cytometry and 38% according to allele-specific oligonucleotide polymerase chain reaction (ASO-PCR). Majority of side effects were related to diarrhea, nausea or fatigue [46].

### Epigenetic agents

Epigenetic dysregulation is a main cause of lymphoma formation and progression. Targeting epigenetic modification mechanisms is a novel approach in treating MCL. Cladribine, a hypomethylating agent that indirectly downregulate DNA methylation, and Vorinostat, a histone deacetylase inhibitor, have been used as one of the combination regimens in treating MCL. A phase I/II trial, combining Vorinostat, Cladribine, and Rituxan, reached a ORR of 97% and CR of 80%, with a 2 year PFS of 70.7% and OS of 86.9% [47]. In other studies combining Velcade, Cladribine, and Rituxan, the ORR and CR for both new and relapsed/refractory MCL were 85% and 77% respectively [48, 49].

### Immunomodulatory Agent

Lenalidomide is an immunomodulatory agent with anti-tumor activities. In various early phase trials, lenalidomide monotherapy in relapsed/refractory setting, could result in an OS of 28–57% and CR of 7.5–36% [50–52]. These trials show median PFS from 4 to 5.7 months. When lenalidomide and bortezomib combination was used in relapsed patients for induction and maintenance therapy, outcomes were not satisfactory with median PFS and OS of 7 months and 26 months respectively, and ORR and CR of 39.6% and 15.1% respectively [53]. These disappointing results were thought to be due to lenalidomide toxicity related dose reduction and inadequate dosing.

When lenalidomide was combined with rituximab in relapsed setting, PFS and OS improvements were noted, with a median of 11.1 months and 24.3 months respectively [54]. Exceptionally high response rate was achieved when lenalidomide was combined with rituximab in induction and maintenance therapy (ORR 92% and CR rate of 64%) in the first line setting. This combination resulted in grade 3/4 neutropenia in 50% patients, 29% of them experiencing grade 3/4 rashes. Lenalidomide therapy also predisposes patients to secondary cancers. When lenalidomide was combined with rituximab, investigators found higher incidence of non-invasive skin cancers though cases of Merkel cell carcinoma and pancreatic cancer were also reported [55]. Although there is clinical benefit of using lenalidomide and rituximab in first line setting, duration of maintenance therapy is not well defined. A 5 years outcome of this combination was presented at 2017 ASH annual meeting, with median follow up of 58 months, 61% evaluable patients had remained in remission. Median PFS was not reached, but estimated 3 and 4 years OS rates were 80.3% and 69.7% respectively [56]. This data highlights that combination therapy of lenalidomide with rituximab in first line setting can result in long-term remission in MCL patients.

Nordic lymphoma Group looked into efficacy of lenalidomide combining with bendamustine and rituximab as a first line treatment in elderly patients (median age 72 years). This study included 6 cycles of induction therapy followed by 52 weeks maintenance therapy with lenalidomide. After completion of induction therapy, 64% patients had CR and 36% were minimum residual disease (MRD) negative. Median follow up for this study was 31 months, with median PFS and OS were 42 months and 53 months respectively [57]. Major limitation of this combination was high incidence of serious infections, which makes this treatment difficult for elder patients.

### Mammalian target of rapamycin (mTOR) inhibitors

Temsirolimus is a specific inhibitor of mTOR kinase, it has been evaluated in refractory/relapsed MCL setting. In a phase III RCT, temsirolimus given 175 mg per week for 3 weeks, followed by weekly dose of 75 mg achieved an objective response rate of 22% and PFS of 4.8 months [58]. Different doses regimen of temsirolimus has been used as monotherapy in relapsed/refractory setting with ORR 38–41%, CR 3–3.7% and median OS 12 months [59, 60]. In a phase I study, temsirolimus also showed to be safe and efficacious when combined with bendamustine and rituximab in refractory setting [61]. When another mTOR inhibitor, everolimus was used in refractory setting, the results were not too encouraging as it only showed modest activity in refractory setting. In that study, the ORR was 8.6% (all partial responses), median PFS and median OS of 4.4 months and 16.9 months respectively [62].

### Role of chimeric antigen receptor-engineered T-cells (CART) and Bi-specific T-cells engager (BiTE) therapy

CART offers innovative intervention for MCL patients. CART therapy improves response duration of those refractory/relapsed MCL patients as well [63, 64]. Investigators are modifying various parts of CART therapy to improve the feasibility and efficiency of MCL treatment. One study modified preparatory regimens for CART therapy, another study further optimized CART cells (JCAR-17) [65, 66]. One study even linked chimeric antigen receptor-engineered exosome (CAR-Exo) with membrane fused CD-19 scFV (single chain region of antibody variable region) in order to deliver drug-containing exosome into the lymphoma cells [67]. A multicenter phase 2 study is currently underway to evaluate the role of anti CD-19 CART (axicabtagene ciloleucel) therapy (KTE-C19) in patients with relapsed/refractory MCL (ZUMA-2) [68].

BiTE therapy transiently engages CD3+ T cells with B cells and results in T cell mediated B cell destruction. In a phase I trial for heavily pretreated Non-Hodgkin Lymphoma patients (included 24 MCL patients), Blinatumomab (bispecific CD19/CD3 antibody) showed single agent activity in MCL patients with ORR 71% [69]. A long-term follow-up analysis of 38 patients with relapsed refractory NHL (14 MCL patients), who achieved an objective response to Blinatumomab, showed median overall survival of 1560 days and median progression free survival of 204 days [70]. A recent phase I study presented at 2018 ASH meeting showed clinical efficacy of Mosunetuzumab (bispecific CD20/CD3 antibody) in relapsed refractory NHL (3 MCL patients). Interestingly, responses were also observed in patients thought to be CD20 refractory and who relapsed following CD19 directed CAR-T therapy [71].

Challenges in MCL treatment include pathophysiological variety, high incidence of disease progression and recurrence, shorter disease free interval, advanced patient age and comorbidity. Besides above-mentioned therapies, NK-kB signal pathway blockage studies showed its potential therapeutic significance [72]. The ultimate goal of MCL treatment is to achieve long-term remission without excess toxicities. Developing personalized precise therapeutic strategy is the direction to go.
